# Three Complete Mitochondrial Genomes of *Ocellarnaca* (Orthoptera, Gryllacrididae) and Their Phylogenies

**DOI:** 10.3390/biology14091231

**Published:** 2025-09-10

**Authors:** Ting Luo, Yanting Qin, Xiangyi Lu, Siyu Pang, Xun Bian

**Affiliations:** Key Laboratory of Ecology of Rare and Endangered Species and Environmental Protection (Guangxi Normal University), Ministry of Education, Guilin 541006, China; luot2024@163.com (T.L.); qinyanting2019@163.com (Y.Q.); luxiangyi631@163.com (X.L.); pangsiyu0820@outlook.com (S.P.)

**Keywords:** Gryllacrididae, mitogenome, *Ocellarnaca*, mitochondrial DNA sequence repeats, phylogenetic analysis

## Abstract

The Raspy crickets are an interesting group of nocturnal animals that bear femoral-abdominal stridulation and spin silk from the mouthparts. Gryllacridid classification is the subject of ongoing discussion. Here, we present the first mitogenomic sequences for three *Ocellarnaca* taxa: *O. braueri* (15,597 bp), *O. fuscotessellata* (15,607 bp), and *O. emeiensis* (16,510 bp). Three mitochondrial genomes exhibited the conventional metazoan gene and conserved the characteristic gene order across Gryllacrididae species. Evolutionary selection analyses showed that *atp8* was the least evolutionarily constrained mitochondrial gene, whereas *cox1* was the most conserved across lineages. The three *Ocellarnaca* species harbored 5–8 mitochondrial DNA sequence repeats (mtSSRs), falling within the 1–8 range detected in all analyzed Gryllacrididae species. *Magnigryllacris* and *Ocellarnaca* exhibited higher mtSSR counts than related genera sharing analogous male abdominal apex morphology. Phylogenomic analyses of 35 mitogenomes from 21 Gryllacrididae species supported *Ocellarnaca* as monophyletic and a sister to *Magnigryllacris* (bootstrap = 100%), with *O. fuscotessellata* resolved as sister to the clade (*O*. sp. + (*O*. *emeiensis* + *O*. *braueri*)). This study expands the mitogenomic resources for *Ocellarnaca*, which will facilitate further resolution of phylogenetic reconstruction within this genus and across Gryllacrididae genera.

## Simple Summary

We performed a systematic analysis of mitochondrial genome features in the family Gryllacrididae (also called raspy cricket). The mitochondrial genomes of three species within the genus *Ocellarnaca* were sequenced, revealing 111 simple sequence repeats (SSRs) across 18 species. Phylogenetic trees based on maximum likelihood and Bayesian inference demonstrated that all species of *Ocellarnaca* clustered together, forming a monophyletic group, with all branches showing statistically robust support values. The mitogenomic resources for the genus *Ocellarnaca* were expanded in this study.

## 1. Introduction

Members of the family Gryllacrididae (also called raspy cricket) [1] are nocturnal animals that hide by day and come out by night. The family contains 972 extant species and 133 genera, primarily distributed in tropical and subtropical regions worldwide [2]. China has 195 species belonging to 30 genera, mainly distributed in southern regions [1,2,3]. As their name suggests, raspy crickets produce a raspy noise during courtship or when threatened, achieved through femoral-abdominal stridulation, wherein the hind femur rubs against pegs on tergites II and III [4]. These animals are also notable for their ability to produce silk from their mouthparts for nest construction, and their exceptional nest-locating capabilities, which ensure that they can return to their shelters easily [5]. However, behavioral, ecological, and applied research on raspy crickets is limited because of their nocturnal habits.

Gryllacrididae classification is the subject of ongoing discussion [1]. Raspy crickets have been traditionally classified into eight distinct types, based on the presence or absence of a median furrow or split on the male ninth abdominal tergite [6]. However, another classification system divided Gryllacrididae into five types based on tegminal venation [7,8]. While the monophyly of Gryllacrididae has been confirmed [9,10], its internal relationships remain unresolved. Observations of vein variation contributed to the identification of three gryllacridid generic groups from Southeast Asia and New Guinea [11]. One year later, a new classification segregated Gryllacrididae into two distinct subfamilies (namely Gryllacridinae and Hyperbaeninae) based on common generic characteristics and distribution [12]. However, in a taxonomic revision, Gryllacridinae was placed under Stenopelmatidae, whereas Hyperbaeninae was regarded as a new synonym of Gryllacridinae [13].

Recent advances in molecular biology and genomics have been crucial in addressing phylogenetic relationships. Mitochondrial genomes (mitogenomes) are effective tools for studying evolution in humans, animals, and plants [14,15,16,17,18,19]. Phylogenomic analyses have provided resolution of the phylogenetic relationships among Orthoptera: (Rhaphidophoridae (Schizodactylidae ((Gryllacrididae (Stenopelmatidae  +  Anostostomatidae))  +  (Prophalangopsidae  +  Tettigoniidae)))) [20]. A recent study also supported the non-monophyletic relationships between the subfamilies Hyperbaeninae and Gryllacridinae based on 13 protein-coding genes (PCGs) [21], aligning with Brunner’s classification but contradicting that of Cadena-Castañeda [12]. Additionally, the monophyly of *Homogryllacris* Liu, 2007, *Furcilarnaca* Gorochov, 2004, *Ultragryllacris* Gorochov & Dawwrueng, 2015, and *Capnogryllacris* Karny, 1937 was recovered [22]. A phylogenetic analysis of Chinese Gryllacrididae species revealed that the genera *Phryganogryllacris* Karny, 1937, *Capnogryllacris*, and *Eugryllacris* Karny, 1937 failed to form monophyletic groups, resulting in six new genera being proposed [1]. The genus *Ocellarnaca* Gorochov, 2004 is defined by a brownish or yellowish-brown color, a median ocellus as large as or slightly wider than the antennal sockets, and the ninth abdominal tergite in males having a pair of lobiform processes, each bearing one spine. *Ocellarnaca* currently includes 17 valid species, but the complete mitogenome of only one species of unknown name has been defined (*Ocellarnaca* sp., MT849269) [23].

Mitochondrial DNA sequence repeats (mtSSRs) are polymorphic DNA motifs characterized by tandemly repeated short units and elevated mutation rates, enabling their wide employment as stable and versatile genetic markers in molecular studies [24,25].

We assembled and annotated the mitochondrial genomes of three species in this investigation, *O. braueri* (Griffini, 1911), *O. emeiensis* Li, Fang, Liu & Li, 2014, and *O. fuscotessellata* (Karny, 1926), and compared and characterized their structure and nucleotide composition. MtSSRs were analyzed across the family Gryllacrididae to identify stable molecular markers. Furthermore, phylogenetic reconstruction was conducted to elucidate the taxonomic position of the three species relative to other Gryllacrididae species.

## 2. Materials and Methods

### 2.1. Sample Acquisition and DNA Extraction

Samples of three *Ocellarnaca* species were collected in 2019 in the field: *O. braueri* (GXNUXZ5) and *O. fuscotessellata* (GXNUXZ7) from Maoershan, Xing’an (25.882° N, 110.490° E, 12 July 2019, collected by Xun Bian), and *O. emeiensis* (GXNUXZ34) from Rongshui (25.075° N, 109.283° E, 1 August 2019, collected by Wei Bin) (Figure S1). All locations were located in the Guangxi Zhuang Autonomous Region of China. Field-collected specimens were promptly fixed in 100% ethanol and transferred to the laboratory for storage at −18 °C. Subsequently, DNA extraction was performed on hind femur muscle samples using the TIANamp Genomic DNA Kit (Tiangen, Beijing, China) in accordance with the supplier’s protocol. The voucher specimens were deposited at Guangxi Normal University, Guilin, China.

### 2.2. Next-Generation Sequencing, Genomic Assembly and Analysis

Total DNA was high-throughput sequenced on an Illumina NovaSeq platform, provided by Beijing Berry Genomics Co., Ltd. (Beijing, China). High-quality clean reads were obtained by filtering raw paired-end data through CLC Genomics Workbench 12 (CLC Bio, Aarhus, Denmark) with default parameters [26]. Processed reads were aligned to the mitogenomic reference sequence with *Homogryllacris anelytra* Shi, Guo & Bian, 2012 (GenBank Accession number NC033998) [10] as a reference. The sequences were assembled using NOVOPlasty v.4.2.1 [27] and annotated by the MITOS web server (http://mitos.bioinf.uni–leipzig.delindex.py, accessed on 10 March 2023) [28]. Mitogenome circular diagrams were generated using the Proksee online platform (accessed on 4 March 2025) [29] and submitted to GenBank with the accession identifiers NC_069863 (*O. fuscotessellata*), NC_069865 (*O. emeiensis*), and NC_069864 (*O. braueri*). Furthermore, the mitogenomes nucleotide composition and codon utilization frequencies of *O. braueri*, *O. fuscotessellata,* and *O. emeiensis* were proven using MEGA v.11.0 [30]. Relative synonymous codon usage (RSCU) was calculated with PhyloSuite v.1.2.3 [31]. AT-skew and GC-skew were calculated using the following formulas: AT-skew = (A − T)/(A + T) and GC-skew = (G − C)/(G + C). The evolutionary rate of each gene was computed using the DnaSP v.6.12.03 [32].

The detection of short mtSSRs was performed using the MIcroSAtellite tool (https://webblast.ipk-gatersleben.de/misa/, accessed on 5 March 2025) [33] on eighteen complete mitogenomes sequenced from Gryllacrididae species, including *O*. *braueri*, *O*. *fuscotessellata*, and *O*. *emeiensis*. The set minimum repeat lengths were as follows: ≥12 for mononucleotides, ≥6 for dinucleotides, ≥4 for trinucleotides, and ≥3 for tetra-, penta-, and hexanucleotides [34]. An interruption of 0 was assumed between two microsatellites. To standardize the comparative analysis across genomes of varying sizes, SSR distributions were normalized to 1 kilobase (kb) genomic intervals using two metrics: relative abundance (RA): the total number of SSR motifs per kb of genome, and relative density (RD): the total length of SSR sequences per genome nucleotide.

### 2.3. Phylogenetic Analysis

Phylogenetic analyses were conducted, including the three recently obtained mitogenomes of *Ocellarnaca*, and 32 mitogenomes belonging to 21 species of Gryllacrididae available in the National Center for Biotechnology Information (NCBI) GenBank database (Table S1). Four additional mitogenomes were employed as outgroups (Table S1): *Troglophilus neglectus* Krauss, 1879 NC_011306 [35] and *Rhaphidophora quadrispina* Liu & Bian, 2021 NC_067624 [36] from Rhaphidophoridae, *Ammopelmatus fuscus* (Haldeman, 1852) NC_028058 [37] from Stenopelmatidae, and *Tarragoilus diuturnus* Gorochov, 2001 NC_021397 [38] from Prophalangopsidae.

Thirteen PCGs and two rRNAs were aligned using MAFFT v7.505 [39]. Although MAFFT alignment does not account for structural constraints, the FFT-NS-1 (fast) algorithm is effective for most global rRNA alignments, delivering rapid processing and accurate results for related species. The alignments of 35 genes from 21 Gryllacrididae species were concatenated to generate datasets for four taxa: (1) the PCG12 matrix with 7642 bp, including the first and second codon positions of PCGs; (2) the PCG123 matrix with 11,463 bp, including all three codon positions of PCGs; (3) the PCG12+2R matrix with 10,007 bp, including the first and second codon positions of PCGs and two rRNA genes; and (4) the PCG123+2R matrix with 13,828 bp, including all three codon positions of PCGs and two rRNA genes. Heterogeneity analysis was performed using AliGROOVE v.1.08 [40]. PhyloSuite v.1.2.3 [31] was used to construct phylogenetic trees using the Bayesian inference (BI) and maximum likelihood (ML) methods. For ML analysis, IQ-TREE v2.2.0 [41] was used with 1000 replicates, where the optimal nucleotide substitution model was identified as TIM + F + I + G4 [42] (Table S2). Bayesian inference was performed in MrBayes v3.2.7a [43] through two parallel MCMC simulations. The optimal substitution model (GTR + F + I + G4), identified by ModelFinder [42] under the BIC framework (Table S2), involves 2 million generations with sampling every 1000 generations. After discarding the first 25% of generations as burn-in, a consensus topology was reconstructed from the remaining trees, with nodal support assessed via posterior probabilities. The BI analysis was considered to have achieved convergence when the average standard deviation of split frequencies (ASDSF) fell below 0.01 [42]. We employed the iTOL online tool (https://itol.embl.de, accessed 7 May 2025) to enhance topological clarity [44].

## 3. Results and Discussion

### 3.1. Genome Structure and Composition of the Three Raspy Cricket Species

**Figure 1:**
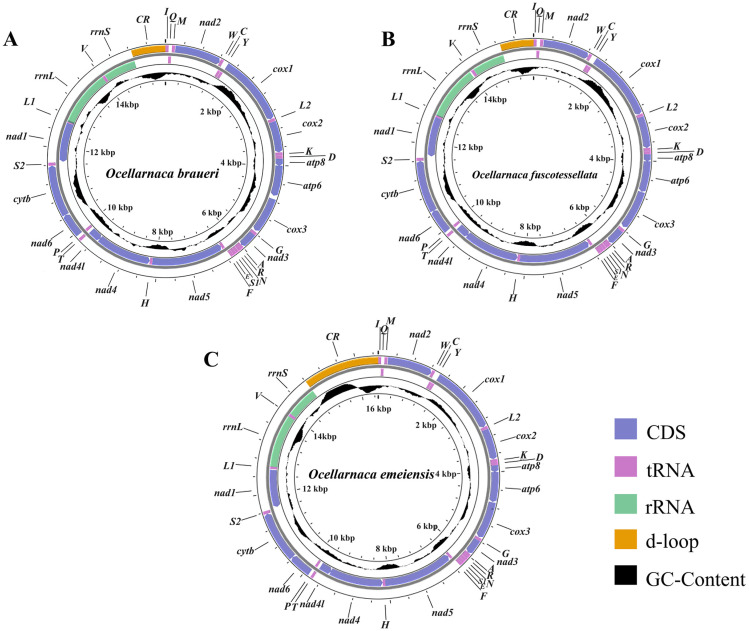
Genome circle map of three *Ocellarnaca* species. (**A**): *O. braueri*; (**B**): *O. fuscotessellata*; and (**C**): *O. emeiensis.* The various gene regions are represented in distinct hues in the depicted genetic sequence. Twenty-two tRNAs are presented through their respective amino acid abbreviations. The J-strand is shown on the outer ring, while the N-strand is displayed on the inner ring. The GC content is graphically represented in black.

The complete mitogenomes of *O. braueri*, *O. emeiensis,* and *O. fuscotessellata* had lengths of 15,597 bp, 16,510 bp, and 15,607 bp, respectively. Three mitogenomes exhibited the typical 37 genes, comprising 13 PCGs, 22 tRNAs, and 2 rRNAs, along with a control region. Gene distribution showed 23 genes encoded on the J-strand and 14 genes on the N-strand (Figure 1).

**Figure 2:**
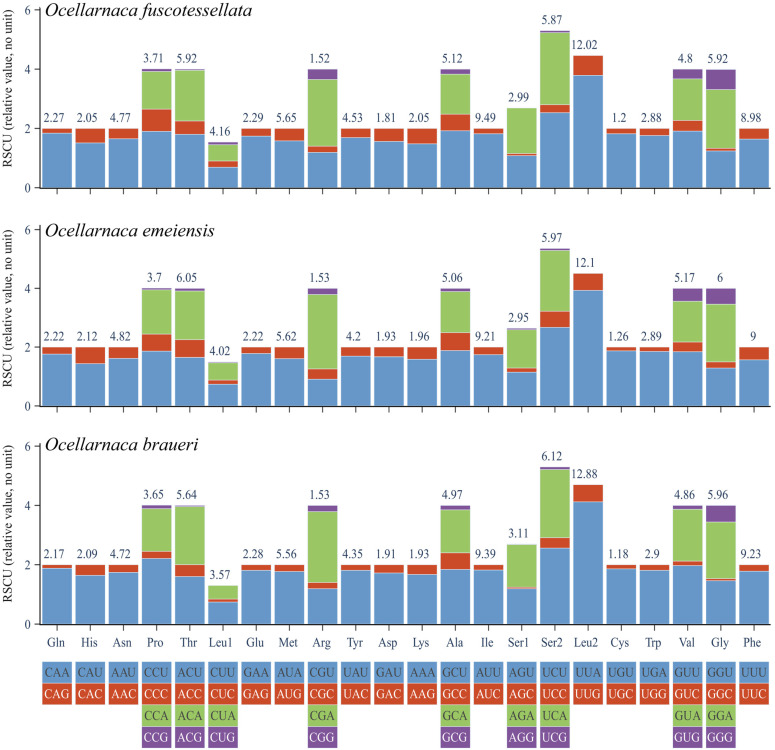
Relative synonymous codon usage (RSCU) of the three complete *Ocellarnaca* mitogenomes. Note, RSCU is a relative value calculated from codon usage counts, which labels it as ‘(relative value, no unit)’ to indicate that it is a relative metric.

The three species exhibited a similar nucleotide composition, with a strong bias towards AT base composition, ranging from 71.87% to 74.68% (mean value = 73.22%), consistent with the pattern observed in most orthopteran insects [10,45,46,47]. RSCU analysis identified UUA (Leu2) as the most preferred codon, with RSCU value varying from 12.02 to 12.88. By contrast, UGU (Cys) was the least used, with the highest values utilizing U and A, meaning an overall skew toward A and T in the three mitogenomes (Figure 2).

### 3.2. Protein-Coding Genes and Evolutionary Rates

Nine PCGs (*nad2*, *cox1*, *cox2*, *atp8*, *atp6*, *cox3*, *nad3*, *nad6,* and *cytb*) were encoded on the J-strand, whereas four PCGs (*nad5*, *nad4*, *nad4l*, and *nad1*) were encoded on the N-strand (Figure 1). Among all PCGs, *nad5* exhibited the maximum length (1735–1738 bp), while *atp8* was the least (159 bp). Eight PCGs (*nad2*, *cox1*, *cox2*, *atp8*, *atp6*, *nad4l*, *nad3*, and *cytb*) exhibited complete length conservation across the three species. Mitochondrial initiation codons predominantly utilized the ATN, except for *nad1*, where TTG was identified in *O. fuscotessellata* (Table S3) and *O. braueri* (Table S4). Although TAA served as the canonical termination codon for most PCGs, truncated termination signals (T/TA) were observed in *cox2*, *nad4*, and *nad5* of *O. braueri and O. emeiensis* (Tables S4 and S5, respectively). This incomplete termination pattern was also conserved in *cox2* and *nad5* of *O. fuscotessellata* (Table S3).

**Figure 3:**
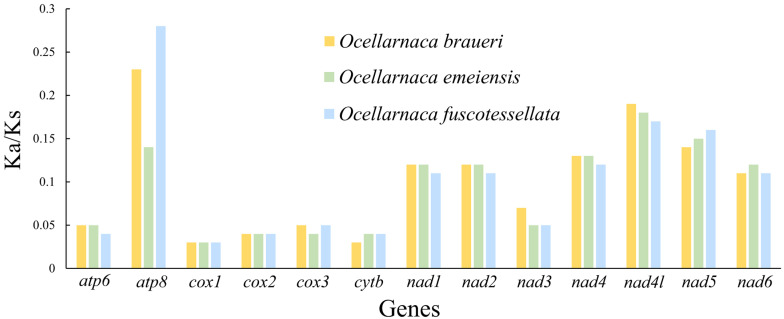
The Ka/Ks ratios of 13 PCGs of the three complete *Ocellarnaca* mitogenomes. Note, Ka: nonsynonymous substitution rate, Ks: the synonymous substitution rate.

We performed Ka/Ks analysis of the 13 PCGs of the mitogenomes to investigate the evolutionary selection constraints of the three *Ocellarnaca* species (Figure 3). All PCGs exhibited Ka/Ks values less than 1, demonstrating purifying selection across the genomes [48]. Among them, *atp8* of *O*. *braueri* and *O*. *fuscotessellata* exhibited the most elevated Ka/Ks ratios, indicating the relaxed purifying selection. By contrast, *cox1* of all three species had the lowest Ka/Ks ratio, suggesting that harmful mutations were eliminated by purifying selection to preserve the integrity of the complex core subunits [49,50].

### 3.3. RNAs and Control Regions

The total lengths of 22 tRNAs of the three species ranged between 1451 bp (*O. fuscotessellata*; Table S3) and 1455 bp (*O. braueri* and *O. emeiensis*; Tables S4 and S5, respectively), among the 22 tRNA genes spanning from 62 to 74 bp. Fourteen tRNA genes (*trnI*, *trnM*, *trnW*, *trnL2*, *trnK*, *trnD*, *trnG*, *trnA*, *trnR*, *trnN*, *trnS1*, *trnE*, *trnT*, and *trnS2*) were J-strand encoded, and eight (*trnQ*, *trnC*, *trnY*, *trnF*, *trnH*, *trnP*, *trnL1*, and *trnV*) were N-strand located. Except for *trnS1*, the tRNAs of the three mitogenomes lacked a dihydrouridine (DHU) arm, forming a typical cloverleaf structure common in metazoans [51,52,53]. Moreover, 20 wobble base pairs (G-U) were found in *O. braueri*, 24 in *O. fuscotessellata,* and 23 in *O. emeiensis*. The rRNAs (*12S* and *16S*) were transcribed from the N-strand, exhibiting size variations from 1299 to 1355 bp, located at a conserved position between *trnL1* and *trnV*. The length of *12S* rRNA was 774–776 bp, located between *trnV* and CR. The length heterogeneity (726–1703 bp) in the regulatory regions located at the *12S* rRNA-*trnI* junction. Thirty-five SSR loci are present in the control region, among which 13 exhibited mutations (Table S6), suggesting that dynamic changes in repetitive sequences may represent one of the key factors contributing to length variation in this region.

### 3.4. Mitochondrial Microsatellites (mtSSRs)

To conduct mtSSR analysis for the whole mitogenomes, we used the three circular mitogenomes that contained the control regions. The mitogenomes of *O. braueri*, *O. fuscotessellata*, and *O. emeiensis* contained 5–8 mtSSRs, a range that fell within the range of 1–8 mtSSRs observed across all 18 analyzed Gryllacrididae species. *O*. *braueri* and *O*. *fuscotessellata* had the highest number of mtSSRs and presented 8 mtSSRs each. By contrast, *O. emeiensis* had only five mtSSRs, of which one was located in *trnD* and another in the control region (Table 1). A total of 111 mtSSRs were found, representing less than 1% of the mitogenomes analyzed and consistent with nuclear genome microsatellite densities (0.02–3.1%) in insects. A total of 49 SSR mutations were detected in this study, suggesting their potential utility as SSR markers for further investigation (Table S6). Analysis revealed variations in the relative abundance of different SSRs across genera. The average number of mtSSRs in the genera *Magnigryllacris* Li, Yin & He, 2024 and *Ocellarnaca* was higher than in other genera with a similarly shaped median ocellus and a male abdominal apex split along the midline (Table 2).

**Figure 4:**
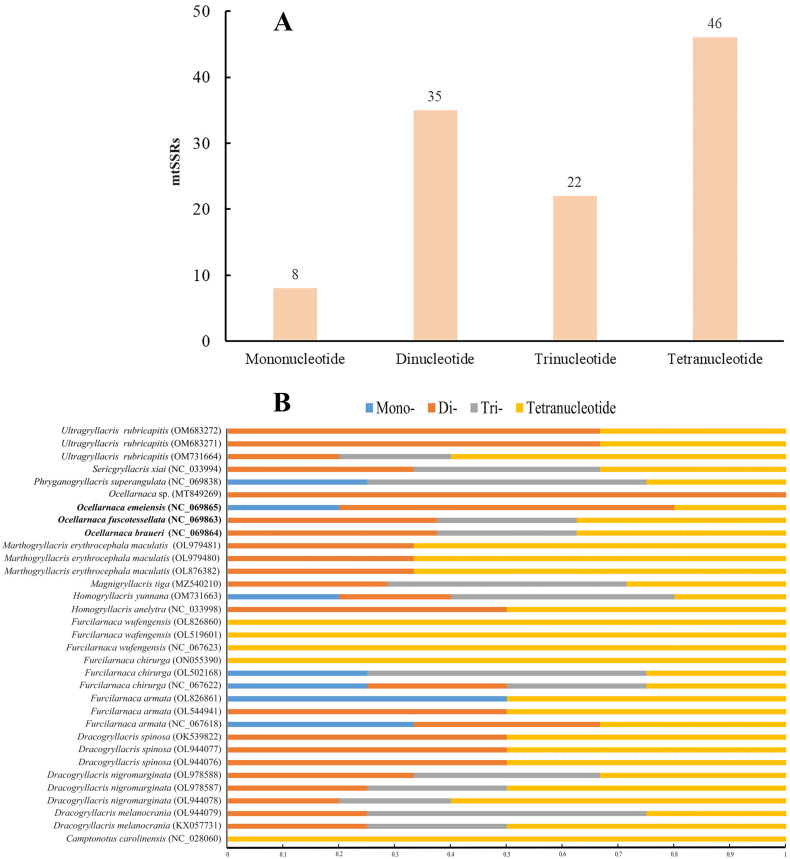
(**A**): The mitochondrial DNA sequence repeats in 18 Gryllacrididae species. (**B**): Relative percentage of four sizes of mtSSRs in 18 Gryllacrididae mitogenomes. The percentages of mono-, di-, tri-, and tetranucleotides are shown in different colors. The Gryllacrididae species are shown to the left.

The RA and RD of the SSRs showed marked variation across mitogenomes. RA ranged from 0.062 mtSSR/kb (*Furcilarnaca wufengensis* Bian, Shi & Guo, 2013, OL519601) to 0.513 mtSSR/kb (*Ocellarnaca emeiensis,* NC069865), and RD spanned from 0.757 bp/kb (*Marthogryllacris erythrocephala maculatis* (Liu, Lu & Bian, 2022) OL979481) to 6.575 bp/kb (*Dracogryllacris spinosa* (Li, Liu & Li, 2014) OL944077) (Table 2). When comparing the number of SSRs within genomes, we found that the percentages of di- (31.5%) and tetranucleotide SSRs (41.4%) were higher than mono- (7.2%) and trinucleotide (19.8%) (Figure 4).

**Figure 5:**
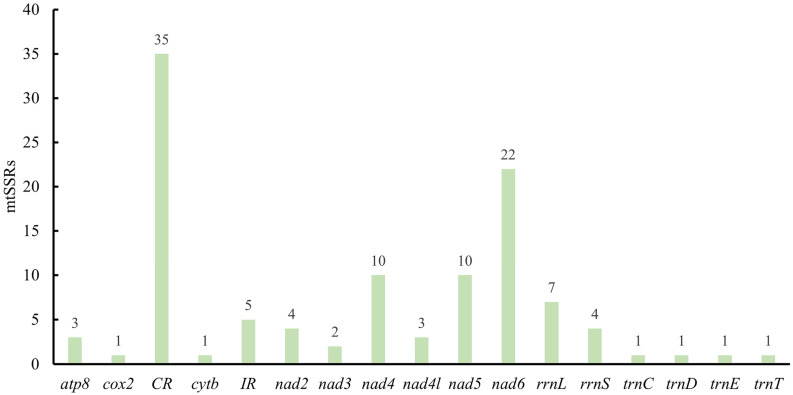
Location of mtSSRs in the mitogenomes of 18 Gryllacrididae species.

Among the identified mtSSRs, 50.5% were localized within PCGs (with the *nad6*, *nad4,* and *nad5* exhibiting the highest mtSSR contents), 45% in non-coding regions (including tRNAs, rRNAs, and control regions), and 4.5% in the intergenic spacer region (IR). The highest abundance of microsatellites corresponded to *nad6* (19.8%), followed by *nad4* (9%) and *nad5* (9%) (Figure 5). A substantial proportion of the coding-region mtSSRs in Gryllacrididae were found in Nicotinamide adenine dinucleotide (NADH) dehydrogenase genes involved in the electron transport system. As a critical redox cofactor, NADH mediates fundamental biological processes, including cellular redox homeostasis, bioenergetic metabolism, mitochondrial bioenergetics, transcriptional regulation, and cell signaling cascades, with its redox couples being indispensable for maintaining physiological functions [54,55,56].

### 3.5. Substitution Saturation and Heterogeneity Analysis

**Figure 6:**
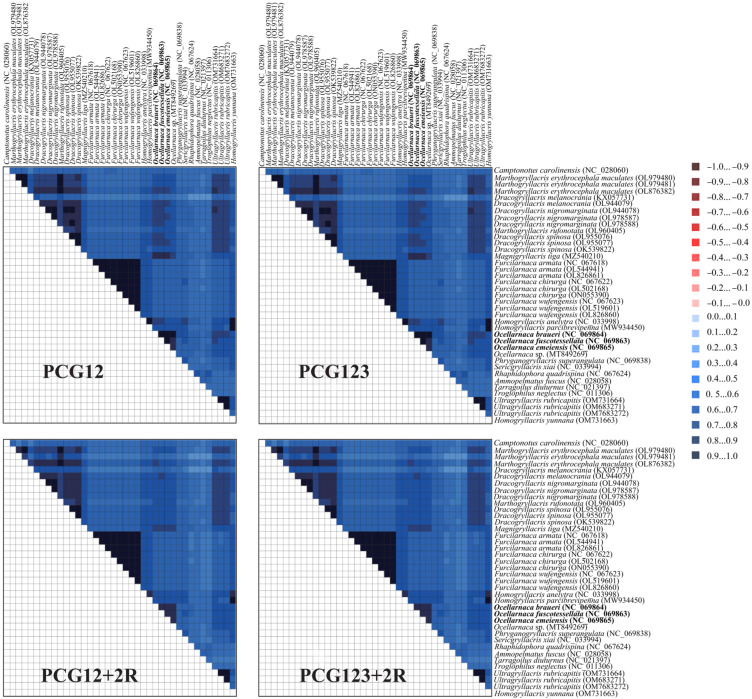
Evolutionary rate heterogeneity among the four Gryllacrididae datasets was assessed using AliGROOVE similarity analysis. The algorithm computes pairwise comparison scores ranging from −1 (red; indicating significant evolutionary rate divergence) to +1 (blue; representing perfect rate congruence).

Heterogeneity among the four datasets (PCG12, PCG123, PCG12+2R, and PCG123+2R) indicated that the PCG12 and PCG123 datasets were unsaturated and had stronger phylogenetic signals. Therefore, they were more suitable for phylogenetic relationships analysis (Figure 6).

### 3.6. Phylogenetic Analysis

**Figure 7:**
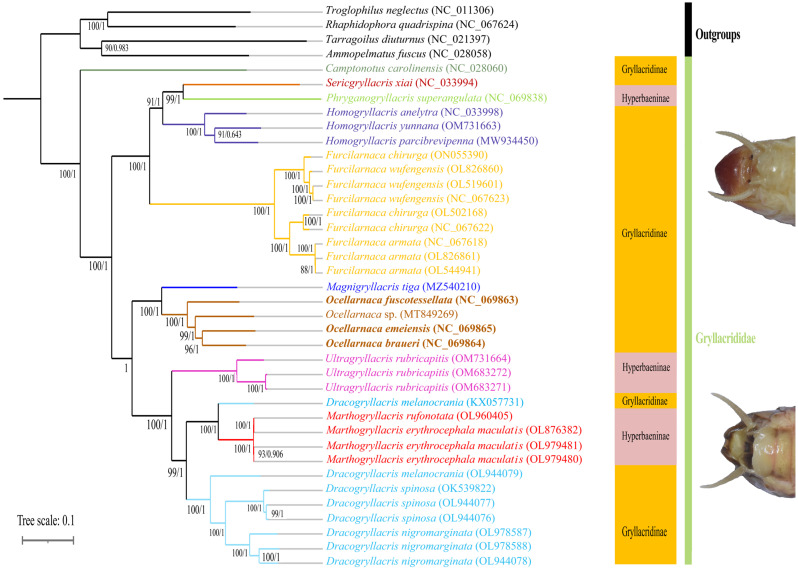
Phylogenetic tree of Gryllacrididae inferred from the maximum likelihood (ML) and Bayesian inference (BI) analyses of the PCG123. Numbers on the branches show bootstrap values. Map the morphological characteristics, the presence or absence of a median groove or midline division on the male ninth abdominal tergite across the two clades onto the phylogenetic tree.

Both ML and BI analyses yielded identical tree topologies, supporting the monophyly of *Ocellarnaca* (ML and BI analyses of PCG123 in Figure 7; PCG12, PCG123+2R, and PCG12+2R analyses in Figures S2, S3, and S4, respectively). Within *Ocellarnaca, O. fuscotessellata* formed a sister group to the clade comprising *O.* sp. and the (*O. emeiensis + O. braueri*) subclade. *Dracogryllacris* Li, Yin & He, 2024 comprises two clades: (A) *Dracogryllacris melanocrania* (Karny, 1929) (KX057731), and (B) the remaining sequences within *Dracogryllacris*. The monophyly of *Homogryllacris*, *Furcilarnaca*, *Ocellarnaca*, *Ultragryllacris*, and *Marthogryllacris* in Gryllacrididae was recovered, with the following phylogenetic relationship: *Camptonotus* + ((((*Sericgryllacris + Phryganogryllacris*) *+ Homogryllacris*) *+ Furcilarnaca*) *+* ((*Magnigryllacris + Ocellarnaca*) + (*Ultragryllacris +* ((clade A *+ Marthogryllacris*) *+* clade B)))), similar to that found in former phylogenetic studies [21,22]. The results of this study, consistent with those of Li [1] and Liu [21], which did not recover the monophyly of Hyperbaeninae and Gryllacridinae (Figure 7), in contrast to the classification proposed by Cadena-Castañeda [12]. Cadena-Castañeda divided Gryllacrididae into Hyperbaeninae and Gryllacridinae based on morphological characters, including body size, wing morphology, tarsal structure, and male genitalia/ovipositor features [12]. However, Liu [21] suggested that the presence or absence of a median groove or midline division on the male ninth abdominal tergite- a character that appears to better reflect Gryllacrididae’s true phylogenetic relationships.

However, our results also indicated that taxonomy at the species level remains partially uncertain. Specifically, *Furcilarnaca chirurga* (ON055390), *F*. *chirurga* (OL502168), and *F*. *chirurga* (OL470659) were not grouped on the same branch (Figure 7), because the *F*. *chirurga* specimen (ON055390) may have been misidentified. Regarding previous studies of Gryllacrididae, few have considered multiple datasets to explore the classification of raspy crickets [21,22]. Molecular or morphological data alone were less likely to provide a complete evolutionary history, even though they could yield unique trees [57,58,59,60]. Future studies could integrate functional morphology with evolutionary models to reveal the evolutionary mechanisms underpinning key adaptive traits in Orthoptera.

### 3.7. Implications for Conservation and Monitoring

Despite its non-threatened status, *Ocellarnaca*, as a common gryllacridid, plays a significant ecological role in forest ecosystems by exerting influence on higher trophic levels through its population dynamics. Its population dynamics may influence higher trophic levels. Due to its specific microhabitat humidity requirements, population fluctuations of *Ocellarnaca* could serve as indicators of forest floor ecosystem health, warranting its inclusion in long-term biodiversity monitoring programs.

## 4. Conclusions

This study reports the complete mitochondrial genomes of three *Ocellarnaca* species (*O*. *braueri*, *O*. *fuscotessellata*, and *O*. *emeiensis*), which exhibit conserved genomic characteristics including size uniformity, AT-rich composition, consistent strand asymmetry (AT-skew and GC-skew), and parallel codon usage patterns. All of the tRNAs maintained complete secondary structures, whereas *trnS1* lacks a DHU arm. A total of 111 Gryllacrididae mtSSRs were identified. Among the three examined species of *Ocellarnaca*, mtSSR counts ranged from 5 to 8. Both *Ocellarnaca* and *Magnigryllacris* exhibited significantly higher mtSSR numbers than lineages with equivalent male abdominal valve lengths. Over 50% of mtSSRs were localized within PCGs, with NADH dehydrogenase genes harboring 91.1% of these PCG-associated repeats. Phylogenetic analysis of 21 Gryllacrididae species based on mitochondrial data reconstructed *Ocellarnaca* as monophyletic, within which three species clustered with *Ocellarnaca* sp., and recovered a sister-group relationship with *Magnigryllacris*. This study supports the need to reevaluate the traditional boundaries between Hyperbaeninae and Gryllacridinae in raspy cricket taxonomy, providing insights for future taxonomic classifications. This study provides fundamental biological data for *Ocellarnaca*, a common yet ecologically significant taxon. While facing no extinction risk, its habitat necessitates inclusion in regional biodiversity monitoring frameworks to safeguard against potential future anthropogenic or environmental disturbances.

## Figures and Tables

**Table 1:** The identified mitogenome sequence repeats (mtSSRs) of three species of *Ocellarnaca*.

Species	mtSSR	Repeats	Size (bp)	Start	End	Location Region
*Ocellarnaca braueri* (NC_069864)	(ATT)_4_	4	12	3876	3887	IR
	(TAA)_4_	4	12	9948	9959	*nad6*
	(TTAT)_3_	3	12	10,124	10,135	*nad6*
	(TAAA)_3_	3	12	13,847	13,858	*rrnL*
	(AATA)_3_	3	12	14,577	14,588	*rrnS*
	(TA)_8_	8	16	15,236	15,251	CR
	(AT)_7_	7	14	15,476	15,489	CR
	(AT)_7_	7	14	15,508	15,521	CR
*Ocellarnaca emeiensis* (NC_069865)	(ATTA)_3_	3	12	3830	3841	*trnD*
	(T)_12_	12	12	16,003	16,014	CR
	(TA)_7_	7	14	16,103	16,116	CR
	(AT)_7_	7	14	16,341	16,354	CR
	(AT)_6_	6	12	16,373	16,384	CR
*Ocellarnaca fuscotessellata* (NC_069863)	(ATT)_4_	4	12	3880	3891	*atp8*
	(AT)_8_	8	16	5576	5591	IR
	(TTAT)_3_	3	12	6321	6332	*trnE*
	(ATT)_4_	4	12	6407	6418	*nad5*
	(TAAA)_3_	3	12	9020	9031	*nad4*
	(TTAT)_3_	3	12	10,141	10,152	*nad6*
	(CA)_7_	7	14	11,678	11,691	IR
	(AT)_7_	7	14	15,472	15,485	CR

**Table 2:** SSR distribution patterns in 18 Gryllacrididae species, with quantitative characterization of repeat number, total length, relative abundance, and relative density.

Species	Kb	Total Number of SSR	RA	Total Length of SSR (bp)	RD
*Camptonotus carolinensis* (NC_028060)	15.211	3	0.197225692	50	3.287095
*Dracogryllacris melanocrania* (KX057731)	16.136	4	0.24789291	36	2.231036
*Dracogryllacris melanocrania* (OL944079)	15.711	4	0.254598689	24	1.527592
*Dracogryllacris nigromarginat* (OL944078)	16.639	5	0.300498828	36	2.163592
*Dracogryllacris nigromarginata* (OL978587)	16.218	4	0.246639536	50	3.082994
*Dracogryllacris nigromarginata* (OL978588)	15.776	3	0.190162272	12	0.760649
*Dracogryllacris spinosa* (OL944076)	15.825	2	0.126382306	60	3.791469
*Dracogryllacris spinosa* (OL944077)	15.817	2	0.126446229	104	6.575204
*Dracogryllacris spinosa* (OK539822)	16.505	2	0.121175401	104	6.301121
*Furcilarnaca armata* (NC_067618)	15.787	3	0.190029771	26	1.646925
*Furcilarnaca armata* (OL544941)	15.830	2	0.126342388	48	3.032217
*Furcilarnaca armata* (OL826861)	15.677	2	0.127575429	12	0.765453
*Furcilarnaca chirurga* (NC_067622)	15.454	4	0.258832665	24	1.552996
*Furcilarnaca chirurga* (OL502168)	15.441	4	0.25905058	12	0.777152
*Furcilarnaca chirurga* (ON055390)	15.550	1	0.064308682	24	1.543408
*Furcilarnaca wufengensis* (NC_067623)	15.954	1	0.062680206	38	2.381848
*Furcilarnaca wufengensis* (OL519601)	16.022	1	0.062414181	26	1.622769
*Furcilarnaca wufengensis* (OL826860)	15.478	1	0.06460783	26	1.679804
*Homogryllacris anelytra* (NC_033998)	15.706	2	0.12733987	60	3.820196
*Homogryllacris yunnana* (OM731663)	16.209	5	0.308470603	48	2.961318
*Magnigryllacris tiga* (MZ540210)	15.513	7	0.451234449	64	4.125572
*Marthogryllacris erythrocephala maculatis* (OL876382)	16.163	3	0.185609107	44	2.722267
*Marthogryllacris erythrocephala maculatis* (OL979480)	16.171	3	0.185517284	86	5.318162
*Marthogryllacris erythrocephala maculatis* (OL979481)	15.845	3	0.189334175	12	0.757337
***Ocellarnaca braueri*** **(NC_069864)**	15.597	8	0.512919151	50	3.205745
***Ocellarnaca*** ***emeiensis* (NC_069865)**	16.510	5	0.512590504	38	2.301635
***Ocellarnaca*** ***fuscotessellata* (NC_069863)**	15.607	8	0.30284676	40	2.562953
*Ocellarnaca* sp. (MT849269)	16.157	3	0.185678034	38	2.351922
*Phryganogryllacris superangulata* (NC_069838)	15.976	4	0.250375563	36	2.253380
*Sericgryllacris xiai* (NC_033994)	15.876	3	0.188964475	38	2.393550
*Ultragryllacris rubricapitis* (OM731664)	15.558	5	0.321378069	66	4.242191
*Ultragryllacris rubricapitis* (OM683271)	16.625	3	0.180451128	60	3.609023
*Ultragryllacris rubricapitis* (OM683272)	15.766	3	0.190282887	12	0.761132

## Data Availability

The original data presented in the study are openly available in the National Center for Biotechnology Information (NCBI, https://www.ncbi.nlm.nih.gov/, accessed on 16 January 2025) genome database.

## Supplementary Materials

The following supporting information can be downloaded at: https://www.mdpi.com/article/10.3390/biology14091231/s1, Figure S1: Ecological photos of three *Ocellarnaca* species; Figure S2: Phylogenetic tree of Gryllacrididae inferred from the ML and BI analyses of the PCG12; Figure S3: Phylogenetic tree of Gryllacrididae inferred from the ML and BI analyses of the PCG123+2R; Figure S4: Phylogenetic tree of Gryllacrididae inferred from the ML and BI analyses of the PCG12+2R; Table S1: List of mitogenomes used for phylogenetic analysis. Table S2: The best-fit partition model of ML and BI analyses; Table S3: Mitochondrial genome content, length, and codon information of *Ocellarnaca fuscotessellata*; Table S4: Mitochondrial genome content, length, and codon information of *Ocellarnaca braueri*; Table S5: Mitochondrial genome content, length, and codon information of *Ocellarnaca emeiensis*; Table S6: Detailed information on mtSSR mutations.
