# Supplementary material for: Three Complete Mitochondrial Genomes of Ocellarnaca (Orthoptera, Gryllacrididae) and Their Phylogenies
Source: Biology (Basel). 2025 Sep 10;14(9):1231. doi: 10.3390/biology14091231 (PMC12467625; doi:10.3390/biology14091231)
Supplement: Supplementary file 1 [file biology-14-01231-s001.zip › Table S1. List of mitogenomes used for phylogenetic analysis..pdf]

Table S1. List of mitogenomes used for phylogenetic analysis.

subfamily	Species name (NCBI)	Accession Number	Species name (current classification system)
Troglophilinae	*Troglophilus neglectus* [1]	NC_011306	
Stenopelmatinae	*Stenopelmatus fuscus* [2]	NC_028058	*Ammopelmatus fuscus* [12]
Rhaphidophorinae	*Rhaphidophora quadrispina* [3]	NC_067624	
Prophalangopsinae	*Tarragoilus diuturnus* [4]	NC_021397	
Gryllacridinae	*Camptonotus carolinensis* [2]	NC028060	
	Phryganogryllacris xiai [5]	NC033994	*Sericgryllacris xiai* [13]
	Homogryllacris anelytra [5]	NC033998	
	Homogryllacris yunnana [6]	OM731663	
	Homogryllacris parcibrevipenna [7]	MW934450	
	Furcilarnaca chirurga [8]	ON055390	
	Furcilarnaca wufengensis [9]	OL826860	
	Furcilarnaca wufengensis [9]	OL519601	
	Furcilarnaca wufengensis [9]	NC067623	
	Furcilarnaca chirurga [9]	OL502168	
	Furcilarnaca chirurga [9]	NC067622	
	Furcilarnaca armata [9]	NC067618	
	Furcilarnaca armata [9]	OL826861	
	Furcilarnaca armata [9]	OL544941	
	Eugryllacris tiga [10]	MZ540210	*Magnigryllacris tiga* [13]
	Ocellarnaca fuscotessellata	NC069863	
	Ocellarnaca sp. [11]	MT849269	
	Ocellarnaca nigra	NC069865	***Ocellarnaca emeiensis*** [14]
	Ocellarnaca braueri	NC069864	
	Capnogryllacris melanocrania [5]	KX057731	*Dracogryllacris melanocrania* [13]
	Capnogryllacris melanocrania [9]	OL944079	*Dracogryllacris melanocrania* [13]
	Dracogryllacris spinosa [9]	OK539822	
	Dracogryllacris spinosa [9]	OL944077	
	Dracogryllacris spinosa [9]	OL944076	
	Dracogryllacris nigromarginata [9]	OL978587	
	Dracogryllacris nigromarginata [9]	OL978588	
	Dracogryllacris nigromarginata [9]	OL944078	
Hyperbaeninae	*Phryganogryllacris superangulata*	NC_069838	
	Ultragryllacris pulchra rubricapitis [6]	OM731664	*Ultragryllacris rubricapitis* [15]
	Ultragryllacris pulchra rubricapitis [6]	OM683272	*Ultragryllacris rubricapitis* [15]
	Ultragryllacris pulchra rubricapitis [6]	OM683271	*Ultragryllacris rubricapitis* [15]
	Marthogryllacris rufonotata [9]	OL960405	
	Marthogryllacris erythrocephala maculates [9]	OL876382	
	Marthogryllacris erythrocephala maculates [9]	OL979481	
	Marthogryllacris erythrocephala maculates [9]	OL979480	
