# Supplementary material for: Three Complete Mitochondrial Genomes of Ocellarnaca (Orthoptera, Gryllacrididae) and Their Phylogenies
Source: Biology (Basel). 2025 Sep 10;14(9):1231. doi: 10.3390/biology14091231 (PMC12467625; doi:10.3390/biology14091231)
Supplement: Supplementary file 1 [file biology-14-01231-s001.zip › Table S2 The best-fit partition model of ML and BI analyses .pdf]

Table S2.1. The best-fit partition model of BI analyses for PCG123+2R.

Subset partitions	Best model
P1: (*atp6*_mafft_*cox1*_mafft_*nad3*_mafft)	GTR+F+I+G4
P2: (*atp8*_mafft_*nad2*_mafft)	GTR+F+I+G4
P3: (*cox2*_mafft)	GTR+F+I+G4
P4: (*cox3*_mafft)	GTR+F+I+G4
P5: (*cytb*_mafft)	GTR+F+I+G4
P6: (*nad1*_mafft)	GTR+F+I+G4
P7: (*nad4l*_mafft_*nad4*_mafft_*nad5*_mafft)	GTR+F+I+G4
P8: (*nad6*_mafft)	GTR+F+I+G4
P9: (*rrnL*_mafft)	GTR+F+I+G4
P10: (*rrnS*_mafft)	GTR+F+I+G4

Table S2.2. The best-fit partition model of BI analyses for PCG12+2R.

Subset partitions	Best model
P1: (*atp6*_mafft_*cox1*_mafft_*cox3*_mafft_*nad3*_mafft)	GTR+F+I+G4
P2: (*atp8*_mafft_*nad2*_mafft)	GTR+F+I+G4
P3: (*cox2*_mafft)	GTR+F+I+G4
P4: (*cytb*_mafft)	GTR+F+I+G4
P5: (*nad1*_mafft)	GTR+F+I+G4
P6: (*nad4l*_mafft_*nad4*_mafft_*nad5*_mafft)	GTR+F+I+G4
P7: (*nad6*_mafft)	GTR+F+I+G4
P8: (*rrnL*_mafft)	GTR+F+I+G4
P9: (*rrnS*_mafft)	GTR+F+I+G4

Table S2.3. The best-fit partition model of BI analyses for PCG123.

Subset partitions	Best model
P1: (*atp6*_mafft_*cox1*_mafft_*nad3*_mafft)	GTR+F+I+G4
P2: (*atp8*_mafft_*nad2*_mafft)	GTR+F+I+G4
P3: (*cox2*_mafft)	GTR+F+I+G4
P4: (*cox3*_mafft)	GTR+F+I+G4
P5: (*cytb*_mafft)	GTR+F+I+G4
P6: (*nad1*_mafft)	GTR+F+I+G4
P7: (*nad4l*_mafft_*nad4*_mafft_*nad5*_mafft)	GTR+F+I+G4
P8: (*nad6*_mafft)	GTR+F+I+G4

Table S2.4. The best-fit partition model of BI analyses for PCG12.

Subset partitions	Best model

P1: (*atp6*_mafft_*cox1*_mafft_*nad3*_mafft)	GTR+F+I+G4
P2: (*atp8*_mafft_*nad2*_mafft)	GTR+F+I+G4
P3: (*cox2*_mafft)	GTR+F+I+G4
P4: (*cox3*_mafft)	GTR+F+I+G4
P5: (*cytb*_mafft)	GTR+F+I+G4
P6: (*nad1*_mafft)	GTR+F+I+G4
P7: (*nad4l*_mafft_*nad4*_mafft_*nad5*_mafft)	GTR+F+I+G4
P8: (*nad*6_mafft)	GTR+F+I+G4

Table S2.5. The best-fit partition model of ML analyses for PCG123+2R.

Subset partitions	Best model
P1: (atp6_mafft_cox1_mafft_cox3_mafft_nad3_mafft)	TIM2+F+I+I+R5
P2: (atp8_mafft_nad2_mafft)	GTR+F+I+G4
P3: (cox2_mafft)	TIM2+F+I+G4
P4: (cytb_mafft)	TIM2+F+I+G4
P5: (nad1_mafft)	TIM+F+I+G4
P6: (nad4L_mafft_nad4_mafft_nad5_mafft)	GTR+F+I+I+R4
P7: (nad6_mafft)	TIM3+F+I+G4
P8: (rrnL_mafft)	TIM3+F+I+G4
P9: (rrnS_mafft)	TIM3+F+I+G4

Table S2.6. The best-fit partition model of ML analyses for PCG12+2R.

Subset partitions	Best model
P1: (*atp6*_mafft_*cox1*_mafft_*nad3*_mafft)	TIM2+F+I+G4
P2: (*atp8*_mafft_*nad2*_mafft)	GTR+F+I+G4
P3: (*co*x2_mafft)	TIM2+F+I+G4
P4: (*cox3*_mafft)	TIM2+F+I+G4
P5: (*cytb*_mafft)	TIM2+F+I+G4
P6: (*nad1*_mafft)	TIM+F+I+G4
P7: (*nad4l*_mafft_*nad4*_mafft_*nad5*_mafft)	GTR+F+I+I+R4
P8: (*nad6*_mafft)	TIM3+F+I+G4
P9: (*rrnL*_mafft)	TIM3+F+I+G4
P10: (*rrnS*_mafft)	TIM3+F+I+G4

Table S2.7. The best-fit partition model of ML analyses for PCG123.

Subset partitions	Best model
P1: (*atp6*_mafft_*cox1*_mafft_*nad3*_mafft)	TIM2+F+I+G4
P2: (*atp8*_mafft_*nad2*_mafft)	GTR+F+I+G4
P3: (*cox2*_mafft)	TIM2+F+I+G4

P4: (*cox3*_mafft)	TIM2+F+I+G4
P5: (*cytb*_mafft)	TIM2+F+I+G4
P6: (*nad1*_mafft)	TIM+F+I+G4
P7: (*nad4l*_mafft_*nad4*_mafft_*nad5*_mafft)	GTR+F+I+I+R4
P8: (*nad6*_mafft)	TIM3+F+I+G4

Table S2.8. The best-fit partition model of ML analyses for PCG12.

Subset partitions	Best model
P1: (*atp6*_mafft_*cox1*_mafft_*nad3*_mafft)	TIM2+F+I+G4
P2: (*atp8*_mafft_*nad2*_mafft)	GTR+F+I+G4
P3: (*cox2*_mafft)	TIM2+F+I+G4
P4: (*cox3*_mafft)	TIM2+F+I+G4
P5: (*cytb*_mafft)	TIM2+F+I+G4
P6: (*nad1*_mafft)	TIM+F+I+G4
P7: (*nad4l*_mafft_*nad4*_mafft)	GTR+F+I+I+R3
P8: (*nad5*_mafft)	TIM3+F+I+G4
P9: (*nad6*_mafft)	TIM3+F+I+G4
