# Supplementary material for: Three Complete Mitochondrial Genomes of Ocellarnaca (Orthoptera, Gryllacrididae) and Their Phylogenies
Source: Biology (Basel). 2025 Sep 10;14(9):1231. doi: 10.3390/biology14091231 (PMC12467625; doi:10.3390/biology14091231)
Supplement: Supplementary file 1 [file biology-14-01231-s001.zip › Table S6. Detailed information on mtSSR mutations..pdf]

mtSSR	Start	Location region
AAAT	8193	*nad4*
TTAT	10125	*nad6*
TTA	11054	*cytb*
AT	15638	CR
TA	15397	CR
AT	15662	CR
ATT	3865	*atp8*
ATT	3918	*atp8*
ATT	6397	*nad5*
ATAA	13638	*rrnL*
AAAT	14583	*rrnS*
AT	15401	CR
CTTA	14319	*rrnS*
ATTT	1295	*trnC*
ACT	14483	*rrnS*
ATCA	730	*nad2*
A	6962	*nad5*
TTTA	10124	*nad6*
AT	13049	*rrnL*
ATT	10017	*nad6*
CAT	5871	*nad3*
TAA	15599	CR
AT	5561	IR

TTAT	6307	*trnA*
TAAA	9004	*nad4*
CA	11665	*trnS*
TAA	9949	*nad6*
TAAA	13865	*rrnL*
AATA	14584	*rrnS*
TA	15397	CR
AT	15662	CR
ATTA	3817	*trnD*
T	15296	CR
AAT	6578	*nad5*
AATA	9101	*nad4*
ATCA	730	*nad2*
ATT	9778	*nad4l*
TTAA	13557	*rrnL*
TTTA	15638	CR
TTA	5915	*nad3*
TA	15641	CR
AATA	9089	*nad4*
TA	5558	IR
ATAA	12713	*rrnL*
CTA	15269	CR
CTA	15279	CR
ATT	653	*nad2*

TCAT	3492	*cox2*
ATAA	15157	CR
