# Supplementary figures and images for: Three Complete Mitochondrial Genomes of Ocellarnaca (Orthoptera, Gryllacrididae) and Their Phylogenies
Source: Biology (Basel). 2025 Sep 10;14(9):1231. doi: 10.3390/biology14091231 (PMC12467625; doi:10.3390/biology14091231)

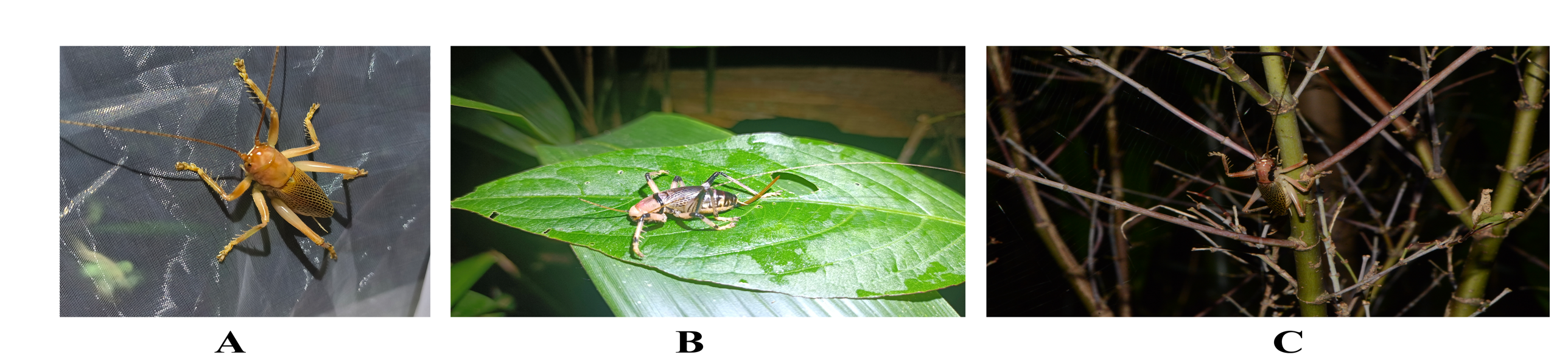

Supplement: Supplementary file 1 [file biology-14-01231-s001.zip › Figure S1. Ecological photos of three Ocellarnaca species.png]
